# Glucocorticoid stress responses of lions in relationship to group composition, human land use, and proximity to people

**DOI:** 10.1093/conphys/cot021

**Published:** 2013-07-12

**Authors:** Scott Creel, David Christianson, Paul Schuette

**Affiliations:** 1 Department of Ecology, Montana State University, 310 Lewis Hall, Bozeman, MT 59717, USA; 2 School of Natural Resources and the Environment, University of Arizona, 325 Biological Sciences East, Tucson, AZ 85721, USA

**Keywords:** Carnivore, glucocorticoid, human–wildlife conflict, Kenya, lion, stress

## Abstract

Large carnivore populations are in global decline, and conflicts between large carnivores and humans or their livestock contribute to low tolerance of large carnivores outside of protected areas. African lions (*Panthera leo*) are a conflict-prone species, and their continental range has declined by 75% in the face of human pressures. Nonetheless, large carnivore populations persist (or even grow) in some areas that are occupied by humans. Lions attain locally high density in the Olkiramatian and Shompole Group Ranches of Kenya's South Rift region, despite residence by pastoralist Maasai people and their sheep, goats, and cattle. We have previously found that these lions respond to seasonal movements of people by moving away from occupied settlements, shifting into denser habitats when people are nearby, and moving into a protected conservation area when people move into the adjacent buffer zone. Here, we examined lion stress responses to anthropogenic activities, using enzyme-linked immunoassay to measure the concentration of faecal glucocorticoid metabolites in 136 samples collected from five lion groups over 2 years. Faecal glucocorticoid metabolite concentrations were significantly lower for lions in the conservation area than for lions in the human-settled buffer zone, and decreased significantly with increasing distance to the nearest occupied human settlement. Faecal glucocorticoid metabolite concentrations were not detectably related to fine-scaled variation in prey or livestock density, and surprisingly, faecal glucocorticoid metabolite concentrations were higher in the wet season, when regional prey abundance was high. Lions coexist with people and livestock on this landscape by adjusting their movements, but they nonetheless mount an appreciable stress response when conditions do not allow them to maintain adequate separation. Thus, physiological data confirm inferences from prior data on lion movements and habitat use, showing that access to undisturbed and protected areas facilitates human–lion coexistence in a broader landscape that is used by people and livestock.

## Abstract (precis)

Large carnivores are in global decline, particularly outside of government protected areas such as national parks. Much is known about the ecology of lions in protected areas, but little is known about situations in which lions coexist with people and livestock. In the Olkiramatian and Shompole area of Kenya's South Rift, lions move among areas with different land uses to avoid direct interactions with people and livestock. When the separation between lions and people is low, lions mount strong glucocorticoid stress responses. These results confirm that access to areas with low disturbance and little interaction with people is important for the conservation of lions outside of national parks.

## Introduction

Large carnivores are in global decline, with strong and pervasive effects on the communities and ecosystems that they have historically occupied (Dill *et al.*, 2003; Estes *et al.*, 2011). As with many other large carnivores, African lions (*Panthera leo*) have lost 75% of their historical range in sub-Saharan Africa (Riggio *et al.*, 2013) as a consequence of human population growth and concomitant pressures, such as habitat loss, prey depletion, trophy hunting, and wire-snare poaching (Creel and Creel, 1997; Woodroffe and Frank, 2005; Packer *et al.*, 2009; Becker *et al.*, 2013a, b; Lindsey *et al.*, 2013). Given that lions sometimes kill humans, they are generally considered ‘conflict prone’ and can be viewed negatively by people living near protected areas (Saberwal *et al.*, 1994; Packer *et al.*, 2005; Woodroffe and Frank, 2005). Attacks on people are rare in most locations, but can become locally common in prey-depleted areas, particularly if a single person sleeps in a crop field to protect it against damage from other species, such as bushpigs (Packer *et al.*, 2005). Although agricultural losses to other species (e.g. elephants, bushpigs, baboons, spotted hyenas, and leopards) are typically much larger (Newmark *et al.*, 1994; Kolowski and Holekamp, 2006; Kissui, 2008), lions are more likely than leopards or spotted hyenas to kill livestock in the daylight hours, which also influences local attitudes toward them (Kissui, 2008). As a consequence of these conflicts, lions are often among the first species to be extirpated locally as human populations grow and intensify land use. Lions also live at relatively low population densities, and lion densities are strongly correlated with the density of ungulate prey (Stander, 1991). Consequently, all remaining large lion populations are restricted to large, ecologically intact, protected areas.

Despite this pattern, it is also clear that much of Africa's wildlife is found outside of areas protected by central governments (Ogutu *et al.*, 2005), and that population trends inside and outside of protected areas sometimes do not differ as much as would be expected, if at all (Western *et al.*, 2009). A substantial fraction of some nations' lions reside outside of governmentally protected areas. For example, an estimated 65% of lions in Kenya persist in unprotected arid rangelands (Chardonnet, 2002). These patterns have motivated efforts to conserve wildlife outside of centrally protected areas or to integrate conservation and development plans, for example by promoting wildlife-based tourism (Newmark and Hough, 2000). For lions and other large carnivores, such programmes might potentially buffer negative human effects on populations within strictly protected areas or augment carnivore populations at the regional scale. Pursuing these potential conservation benefits involves trade-offs, because such programmes can also promote rapid local human population growth near protected areas, exacerbating conservation problems (Wittemyer *et al.*, 2008).

Against this background, it is valuable to understand the differences between areas where lions are extirpated and areas where lions successfully coexist with humans and livestock. A great deal is known about the ecology of lions in protected areas (Schaller, 1972; Orsdol *et al.*, 1985; Stander, 1991; Hanby *et al.*, 1995; Scheel and Packer, 1995) and about the factors that cause human–lion conflict (Ogutu *et al.*, 2005; Woodroffe and Frank, 2005; Kolowski and Holekamp, 2006; Kissui, 2008). Much less is known about the factors associated with successful coexistence, although some populations demonstrate this potential. For example, the Ngorongoro Crater (Tanzania) holds a very dense lion population (22.6–24.8 lions/100 km^2^; Packer *et al.*, 2013) and lies unfenced within the Ngorongoro Conservation Area, with substantial populations of people and livestock. We examined the factors that promote coexistence of people, livestock, and lions in a similar system, the Olkiramatian and Shompole Group Ranches in Kenya's South Rift Valley, between January 2008 and February 2011.

**Figure 1:**
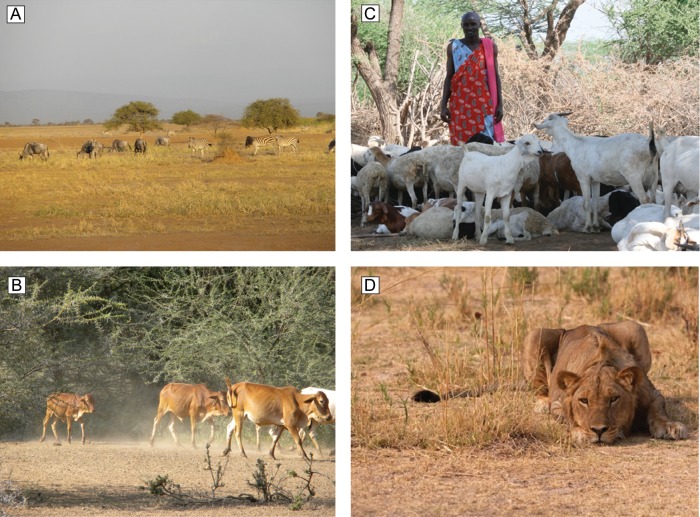
The Olkiramatian and Shompole Maasai Group Ranches include a mosaic of local land uses, including a conservation area with no human settlements that is primarily set aside for wildlife (**A**), grazing areas for livestock (**B**), and human settlement patterns that vary seasonally (**C**). (**D**) Within this matrix of land uses, a population of lions persists at high density, with low rates of reported conflict.

**Figure 2:**
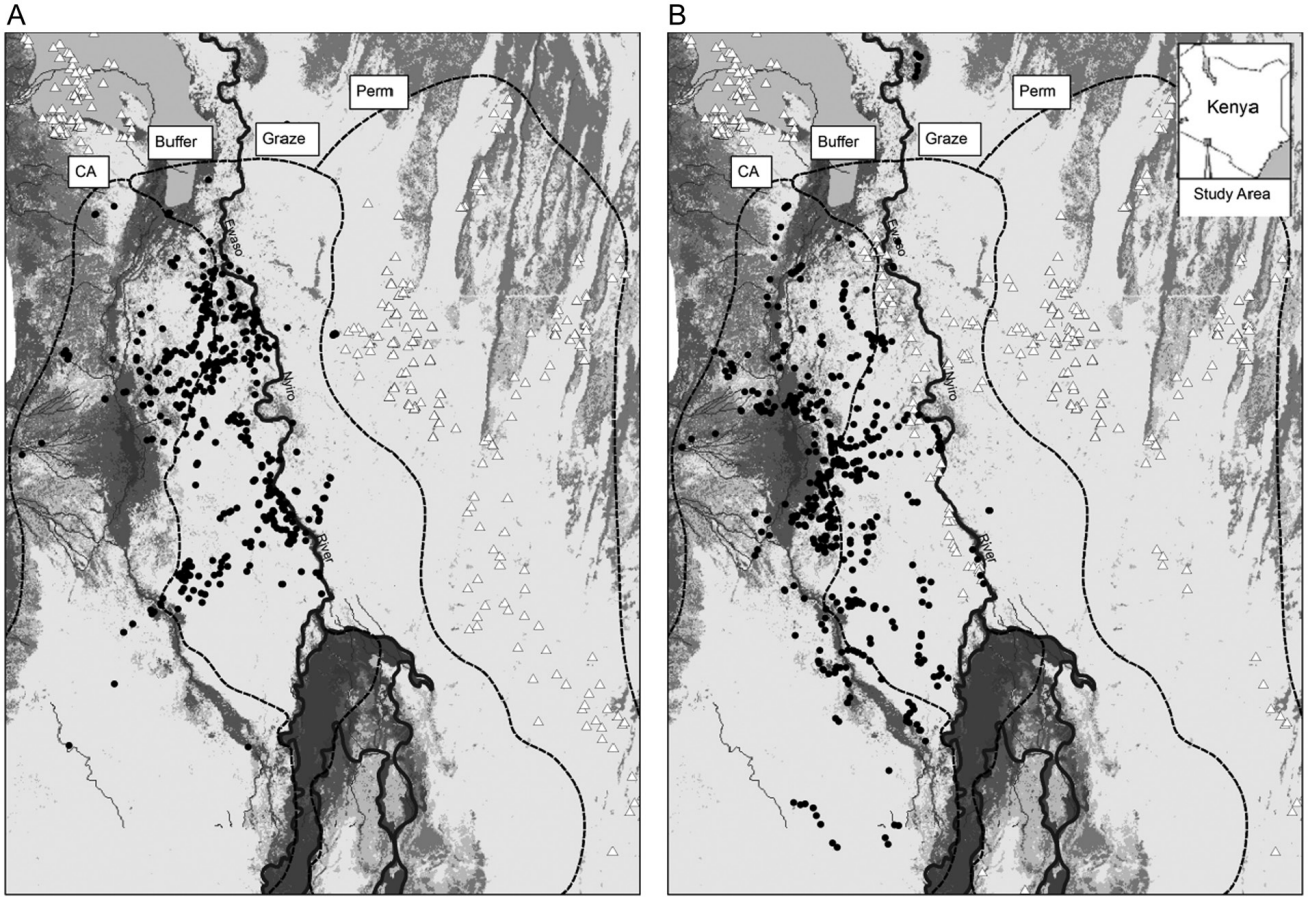
The spatial distribution of land uses in Olkirmatian and Shompole, and lion responses to seasonal variation in human settlement. In the dry season (**A**), people occupy settlements (open triangles) only on the east side of the permanent Ewaso Nyiro river, and lions (filled circles) make heavy use of the buffer zone, including areas immediately west of the river. In the wet season (**B**), people move into seasonal settlements in the buffer zone on the west side of the Ewaso Nyiro, and lions shift westward into the conservation area. Reprinted with permission from Schuette *et al.* (2013a), who provide additional details.

Much of Kenya's arid rangelands were historically used for pastoral grazing, but are now undergoing rapid sedentarization of pastoralist movements, intensification of land use, and habitat fragmentation through privatization of land ownership (Ogutu *et al.*, 2005). In Olkiramatian and Shompole, the community has retained communal ownership of lands and seasonal movements of people and livestock. The Olkiramatian and Shompole governments legally established a Community Conservation Area in 2001, in which there are no human settlements and livestock grazing is allowed only during drought. Thus, the broader landscape remains a mosaic of four land uses that comprise a gradient of human effects (Figs 1 and 2) from year-round human settlement and grazing (‘permanent’), seasonally restricted grazing with no human settlement (‘grazing’), seasonally restricted settlement and grazing (‘buffer’), and no settlement or grazing (‘conservation area’). As has been observed for spotted hyenas in similar circumstances (Boydston *et al.*, 2003; Kolowski and Holekamp, 2009), lions actively respond to the movements of people and livestock to maintain separation (Fig. 2). When people and livestock occupy the buffer zone seasonally, lions retreat into the conservation area, move away from permanent water where livestock aggregate, and increase their use of dense habitats where they are unlikely to be encountered (Schuette *et al.*, 2013a).

By altering their movements and becoming more cryptic, lions in Olkiramatian and Shompole persist with people and livestock and attain a density (13.6 lions/100 km^2^ excluding cubs; Schuette *et al.*, 2013a) comparable to many well-known National Parks (Chardonnet, 2002; Packer *et al.*, 2013). This result does not necessarily imply that coexistence carries no costs.

Here, we used enzyme-linked immunoassay of faecal glucocorticoid metabolites (fGCM) to test for physiological stress responses of lions to anthropogenic stressors. Many experimental studies have shown that psychological stressors can produce strong and persistent stress responses, including activation of the hypothalamic–pituitary–adrenal axis to increase circulating glucocorticoid (GC) concentrations (Weiss, 1970; Sapolsky *et al.*, 2000; Sapolsky, 2002). Psychological stressors generally provoke GC responses if they are uncontrollable or unpredictable in the short term (Weiss, 1970; Sapolsky, 2002), as anthropogenic effects often are. Human disturbance has long been recognized as a stressor (e.g. Döhler *et al.*, 1977; Hannah *et al.*, 1994; Frid and Dill, 2002) and can provoke GC responses in the wild (Wasser *et al.*, 1997; Creel *et al.*, 2002; Van Meter *et al.*, 2009). These GC responses (like behaviour) can serve as a sensitive early indicator of potential effects on demography or population dynamics (Romero and Wikelski, 2001; Frid and Dill, 2002; Pride, 2005; Sheriff *et al.*, 2009). Although abundant data from the biomedical literature suggest that chronic stress is harmful through several physiological pathways (Sapolsky, 1996; McEwen and Wingfield, 2010), it should also be noted that field studies sometimes fail to detect relationships between GC concentrations and fitness components (Bonier *et al.*, 2009; Creel *et al.*, 2009) for a variety of reasons (Dingemanse *et al.*, 2010). By testing for GC responses to human activities and other factors that threaten the persistence of endangered species, conservation physiology can identify situations in which potential negative effects on population dynamics are incipient.

## Materials and methods

### Olkiramatian and Shompole study area

We gathered data from January 2008 to February 2011 in the southern Rift Valley of Kenya on the Olkiramatian and Shompole Maasai Group Ranches, a semi-arid rangeland region with open and closed *Salvadora persica* bushland and *Acacia* woodland, and patches of open grassland. The area receives annual rainfall of 400–600 mm/year, primarily from March to May and from November to December. The permanent Ewaso Nyiro River bisects the study area from north to south, with pronounced differences in land use between the two sides (Fig. 2).

A relatively low-density Maasai community (10 people/km^2^) inhabits Olkiramatian and Shompole, with moderate to high densities of livestock (sheep/goats, 59.1 ± 17.0 individuals/km^2^ and cattle, 15.8 ± 5.7 individuals/km^2^; Schuette *et al.*, 2013a). Despite increasing land sub-division and farmland production on nearby rangeland (Homewood *et al.*, 2009), this region is unfenced in all directions. Although bounded by the Rift Valley escarpment directly to the east, Olkiramatian and Shompole are part of a contiguous trans-boundary arid rangeland ecosystem encompassing >8000 km^2^ that extends across southern Kenya and northern Tanzania. Locally, the Olkiramatian and Shompole landscape is partitioned into four land uses (Fig. 2), including (from west to east): (i) a community conservation area (CA) that is normally unoccupied but can be used as a daytime livestock grazing refuge during severe drought; (ii) a buffer area that is occupied seasonally by Maasai and grazed by livestock during the dry season (September–March); (iii) a grazing area that is seasonally grazed in the wet season (March–September) with no settlements; and (iv) a permanent settlement area that people and livestock occupy throughout the year. Decisions on exactly when and where to settle and graze livestock are made by a committee of Maasai community members. Livestock herds are always accompanied by one or more herdsman and a guard dog during daytime hours (06.00–18.00 h). At night, livestock are invariably kept within a corral built of tightly woven thorn branches (Fig. 1C), positioned at the centre of each Maasai settlement to protect their herds from predation.

We restricted our analysis to data from the conservation area and buffer land use types on the west side of the river, which encompass an area of 250 km^2^. Lions occur on the east side of the river in the grazing and permanent settlement areas, but their occupancy levels are low compared with the west side of the river (Schuette *et al.*, 2013a), so we did not have sufficient data to examine fGCM concentrations of lions east of the river.

The site holds diverse large herbivore and carnivore communities. Densities of the five most common native ungulates [zebra (*Equus quagga*), Grant's gazelle (*Nanger granti*), wildebeest (*Connochaetes taurinus*), impala (*Aepyceros melampus*), and Maasai giraffe (*Giraffa camelopardalis tippelskirchi*)] are comparable to those of many government-protected national parks, despite being outnumbered three to one by livestock (74.9 ± 22.7 livestock/km^2^ and 28.2 ± 9.2 native ungulates/km^2^; Schuette, 2012). The site holds at least 21 carnivore species, including all apex predators, i.e. lion, spotted hyena (*Crocuta crocuta*), leopard (*Panthera pardus*), cheetah (*Acinonyx jubatus*), and African wild dog (*Lycaon pictus*) (Schuette *et al.*, 2013a)

### Field methods and sample collection

From January 2008 to February 2011, we monitored the Olkirmatian and Shompole lion population using radiotelemetry for three resident prides and two male coalitions, and opportunistic data from one pride and two coalitions that were too shy to radio collar. The density of known individuals during the study was relatively constant, with a mean of 13.6 lions/100 km^2^ (excluding cubs <1 year old), which falls toward the upper end of the range of lion densities reported for centrally protected National Parks across Africa. We anaesthetized lions with a combination of medetomidine and ketamine and reversed the effects of medetomidine with atipamezole, under a protocol approved by the MSU Animal Care and Use Committee. We radio collared lions with Telonics MOD-400 VHF transmitters attached to butyl belting collars, with an activity sensor to facilitate observation at night.

We collected 136 fresh faecal samples from four prides and four male coalitions, which held a total of 22 females and 12 males, excluding cubs <1 year old. We collected most samples while following radio-collared lion groups in a four-wheel-drive vehicle using a directional antenna and hand-held VHF receiver, primarily during the night and early morning hours (18.00–08.59 h) when lions were most active. We used red-filtered spotlights and dimmed headlights and followed at a distance that minimized disturbance to lions and potential prey animals, usually ≥100 m. Lions rarely reacted to the vehicle, and we stopped immediately if we detected any reaction. For the interpretation of the results below, we note that any effect of our presence would affect all of the data, and thus would not explain the effects that we observed. To balance the data, we attempted to locate and follow each radio-collared group at least once per week. During each group follow, we recorded a GPS location of the radio-collared group at 5–10 min intervals when lions were actively moving and at every transition from active to stationary. We periodically located radio-collared groups during the daytime hours (09.00–17.59 h) to document daytime resting locations. All daytime locations were spaced a minimum of 24 h apart to ensure that we collected only one daytime location per group per day. All lions were individually recognizable by photographing unique whisker-spot patterns, torn ears, and facial scars (Pennycuick and Rudnai, 1970), but following at night at distances about 100 m, we usually could not ascertain which individual produced a scat sample, although we knew the age class and sex of the defaecating lion for 50 samples.

We used GPS locations at scat collection sites to relate fGCM concentrations to local environmental conditions, using a geographic information system. As previously described in detail by Schuette *et al.* (2013a,b), we used distance sampling on a set of fixed transects to measure spatiotemporal variation across the study site in the distributions of livestock (cattle, sheep, and goats) and native ungulates (wildebeest, zebra, impala, Grant's gazelle, and Maasai giraffe, which collectively constitute >90% of the local ungulate community). We surveyed 16 fixed transects that totalled 69 km. The transects were aligned east–west, not on roads or tracks, systematically spaced at 2 km intervals from north to south across the entire site. We surveyed this set of transects on 21 occasions (6 week intervals), sampling each transect both day and night on each occasion for a total of 2898 km. For each faecal sample, we extracted the density of native prey and livestock from the nearest transect, from the survey that was most closely matched in time. We also used a geographic information system to determine the land use at the location of each sample (conservation area or buffer zone; too few samples were collected from other areas for analysis) and to determine the distance between each faecal sampling site and the nearest occupied human settlement.

We recorded the date of each sample to test for variation in fGCM concentrations between the wet and dry seasons. When possible (*n* = 50), we recorded the identity or age and sex class of the individual sampled, to test for a difference between the sexes. Finally, we related fGCM concentrations to group size and the presence of cubs within a pride, to test whether the social structure of the pride affected GC levels.

### Enzyme-linked immunoassay of faecal glucocorticoid metabolites

When we detected a fresh lion scat, we collected a sample by thoroughly mixing the scat and sub-sampling from several locations to collect 1.8 ml that we stored in an internally threaded cryovial. Samples were kept in a coolbox immediately after collection, transferred to liquid nitrogen within 12 h, and stored in liquid nitrogen until extraction. We extracted steroid hormone metabolites by drying the scat and boiling a known mass of dry faeces in ethanol using published methods that have been described in detail previously (Monfort *et al.*, 1997; Creel *et al.*, 2002). We express fGCM concentrations as nanograms of cortisol immunoreactivity per milligram of dry faeces.

We measured glucocorticoid metabolite concentrations in faecal extracts using an enzyme-linked immunoassay with a cortisol antibody (Enzo Life Sciences ADI-900-071) that has broad cross-reactivity and has previously been validated for assay of faecal extracts in other species (Ezenwa *et al.*, 2012). Antibody binding was closely parallel for a dilution series of cortisol standards and lion faecal extracts diluted from 1:16 to 1:1024. For lion faecal extracts spiked with known amounts of cortisol, recovery was highly accurate (*r*^2^ = 0.997, *b* = 0.998, regression through the origin) for a range from 78.5 to 5000 pg of cortisol added to faecal extracts assayed at working concentration. Assay sensitivity was more than an order of magnitude below the concentration of faecal extracts. The assay was biologically validated by intramuscularly injecting a male lion at Tautphaus Park Zoo (Idaho) with 5 μg/kg of cosyntropin, a synthetic adrenocorticotrophic hormone (ACTH) subunit (Cortrosyn, Amphastar). The mean fGCM concentration following ACTH injection (1059.0 ± 115.5 ng/g dry faeces, *n* = 8) was 177% of pre-ACTH values (600.0 ± 128.0 ng/g dry faeces, *n* = 6, *t* = 2.64, *P* = 0.011), peaking at 243% in the first sample after injection. Based on preliminary analysis of pooled lion faecal extracts, we initially assayed all extracts at 1:30 dilution. If 1:30 dilution did not produce binding near the middle of the standard curve, we re-assayed the extract at a dilution of 1:100 to improve precision. We assayed all samples, standards, and controls in duplicate, including a seven-standard curve, controls and measures of total activity, zero-steroid binding, and non-specific binding on each plate. Intra- and inter-assay coefficients of variation from pooled lion faecal extracts were 10.16 and 13.46, respectively.

The diet of lions varied little during the study, and consisted almost entirely of large ungulates. We detected no association between fGCM concentrations and the composition of scats, as measured by the proportion water or the proportion of indigestible matter.

### Statistical methods

To test what factors affected lion glucocorticoid concentrations, we fitted linear models using the *lm* function in the base ‘stats’ package of R (2002; R Core Development Team, 2008), testing that assumptions were met and assessing goodness of fit by examining Q–Q plots, plots of residuals against predicted values, plots of residuals against leverage, and *r*^2^ adjusted for degrees of freedom. All generalized variance inflation factors were substantially less than five (mean generalized variance inflation factor = 2.00), and we confirmed that regression coefficients with *P* < 0.05 were stable when other regressors were removed from the full model, which included effects of gender, season, group size, presence of cubs, distance to the nearest occupied human settlement, land use (conservation area or buffer zone), local density of prey, local density of cattle, and local density of sheep/goats (combined because they are morphologically and ecologically very similar). We centred and scaled continuous variables (densities of livestock and native ungulate prey, distance to the nearest occupied human settlement, and group size) so that effect sizes could be compared directly.

## Results

Lion fGCM concentrations were significantly correlated with many of the factors that we examined (Table 1), and these factors explained a substantial proportion of the observed variation in fGCM (*r*^2^ = 0.413, *F* = 7.95, d.f. = 128, *P* < 0.000001). With respect to social organization, fGCM concentrations increased with group size (Table 1), as expected if social interactions provoke stress responses (Creel, 2001; Creel *et al.*, 2012), and tended to be higher in males. Glucocorticoid levels were lower in groups that included cubs (Table 1).

**Table 1:** Correlates of lion faecal glucocorticoid metabolite concentrations in Olkiramatian and Shompole

Predictor	Coefficient	Standard error	*t*	*P*-value
Intercept	1226.80	148.68	8.25	<0.000001
Land use = conservation^a^	−305.21	134.85	−2.26	0.0253
Distance to settlement^b^	−272.92	62.99	−4.33	0.0000294
Season = wet^c^	1010.26	285.43	3.54	0.000558
Group size^b^	524.50	95.95	5.47	0.00000023
Cubs = yes^d^	−496.01	166.37	−2.98	0.00343
Sex = female^e^	−133.91	126.53	−1.06	0.292
Sex = male^e^	473.45	243.57	1.94	0.0540
Prey density^b,f^	−112.34	76.72	−1.464	0.146
Cattle density^b,f^	46.55	28.45	1.636	0.105
Sheep/goat density^b,f^	−4.63	10.51	−0.440	0.661

^a^Reference level = buffer zone, so the negative coefficient indicates lower faecal glucocorticoid metabolites (fGCM) in the conservation area.

^b^All continuous variables were centred and scaled prior to analysis so that all effect sizes (coefficients) can be compared directly.

^c^Reference level = dry season, so the positive coefficient indicates higher fGCM in the dry season.

^d^Reference level = cubs absent, so the negative coefficient indicates lower fGCM in groups with cubs present.

^e^Reference level = unsexed, so a negative coefficient indicates low fGCM relative to samples for which sex was not known, and a positive coefficient indicates high fGCM relative to samples for which sex was not known.

^f^These predictors were dropped from the final model, which we identified by backward stepwise selection using a difference in sample-size corrected Akaike Information Criterion of two as the criterion to remain in the model.

**Figure 3:**
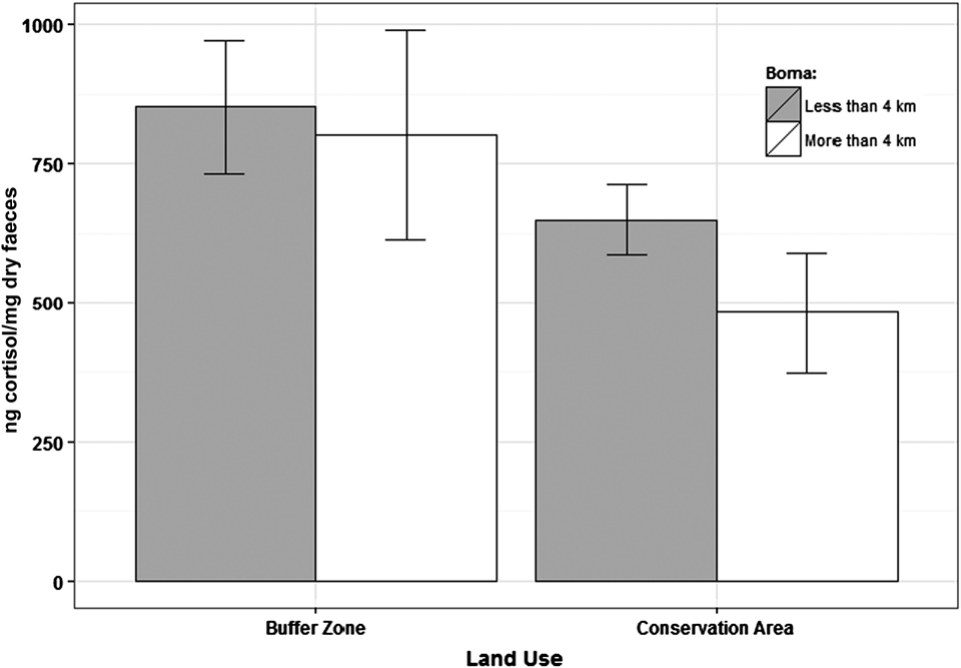
Faecal glucocorticoid metabolite (fGCM) concentrations of lions were higher when lions were in the buffer zone than when they were in the conservation area, and increased with proximity to occupied human settlements.

Two effects on fGCM support the hypothesis that anthropogenic effects increase lion glucocorticoid levels. First, fGCM concentrations decreased significantly with increasing distance to the nearest occupied human settlement (Fig. 3 and Table 1). Second, fGCM concentrations were significantly lower when lions were within the conservation area, relative to fGCM concentrations when they were in the multiple-use buffer zone (Fig. 3 and Table 1). All lion groups that we sampled moved between the conservation area and the buffer zone, so this effect is not confounded with group identity.

Finally, lion fGCM concentrations were not detectably related to short-term variation in the fine-scaled local density of prey, cattle, or sheep and goats (Table 1). Surprisingly, seasonal variation showed that fGCM levels were higher in the wet season, when regional prey abundance was high.

## Discussion

A large proportion of the observed variation in lion glucocorticoid concentrations can be explained by the variables we examined. Faecal glucocorticoid metabolite concentrations were higher in males, in larger groups, and when cubs were absent. All of these effects can be explained by the observation that aggression and agonistic interaction typically increase glucocorticoid secretion (Sapolsky, 2005), as has been reported for several other carnivore species (Creel *et al.*, 1992, 1996; Hacklander *et al.*, 2003; Sands and Creel, 2004; Young *et al.*, 2006). Male lions are more aggressive than females, rates of social interaction correlate with group size, and the presence of cubs can reduce aggression related to mating, because females with cubs do not enter oestrus.

Two results showed that interactions with people and livestock also provoke an increase in glucocorticoid concentrations; both of these responses were detected at a relatively broad spatiotemporal scale, suggesting that they are persistent or chronic. First, fGCM concentrations of lions decreased by 25% when they were within the strictly protected conservation area, relative to fGCM concentrations in the adjacent buffer zone, where people were seasonally resident and livestock grazing was common. Glucocorticoid concentrations also decreased as the distance to the nearest occupied settlement increased, revealing a more fine-scaled response to interactions with people. Like the previous effect, this effect was large, with a 22% decrease in fGCM per kilometre of separation.

Contrary to our expectation, fGCM concentrations were much higher in the wet season. While it is common for glucocorticoid concentrations to vary seasonally (Romero, 2002), we did not expect fGCM concentrations to be higher in the wet season, when the regional density of prey was high (Schuette, 2012). Lions do not breed seasonally, so this common explanation for seasonal variation in GC concentrations (Romero, 2002) also does not explain the pattern we observed. We interpret this result cautiously, because our data were not evenly distributed between the wet and dry seasons. The data were well balanced for the other factors we tested, but most of the samples came from the dry season, so we have limited scope to explain seasonal effects.

At the finest spatiotemporal scale, fGCM concentrations were not detectably related to the local density of prey or livestock. We also interpret this result cautiously. It is possible that lion GC secretion is not related to rates of encounter with livestock (and their attendant herders) or to local prey availability, but it is also true that lion movements can be rapid relative to the 2 km spacing of the transects we used to measure ungulate distributions. Given that we conducted ungulate surveys at 6 week intervals (with a total of 21 surveys), these data provide a good picture of long-term differences between locations in the density of livestock and native ungulates, but they do not provide detailed information on day-to-day variation. It seems probable that the strong effects on fGCM of land use (conservation area vs. buffer zone) and distance to occupied settlements are mediated at least in part by encounters with people and livestock.

The association between lion GC concentrations and human effects parallels prior results on the behavioural responses of lions to human movements (Schuette *et al.*, 2013a). When people moved into the buffer zone, lions shifted their patterns of occupancy to make increased use of the conservation area, increased their use of dense habitats, and increased their distance to permanent water (where human settlements are concentrated; Fig. 2). Collectively, the behavioural and physiological data show that lions move in a manner that reduces the likelihood of interaction with people, and mount a strong stress response to factors that increase the likelihood of interaction with people. It appears that unpredictable or uncontrollable interactions with people are a substantial psychological stressor for lions. Chronic increases in glucocorticoids can provoke a range of pathologies (Sapolsky, 2002, 2005) that could affect the fitness of lions if the frequency or intensity of disturbance becomes too high. For policies both inside and outside of national parks, our results suggest that the provision of refuges from human disturbance is important, even for lions.

The behavioural and physiological data consistently suggest that lions actively avoid direct interactions with people and livestock in this system, and are stressed by contexts that increase the likelihood of direct interaction. The high local lion density and low rates of reported human–lion conflict (Schuette *et al.*, 2013a) suggest that the traditional methods of pastoralist livestock husbandry employed in Olkiramatian and Shompole are a valuable model for coexistence with carnivores outside of centrally protected national parks. Livestock are invariably attended by people while foraging during daylight hours, and even an unarmed young man is a potent deterrent of predation in this context, where opportunities to attack unattended livestock are very rare. Most livestock herds hold <100 animals and can be attended closely. Cattle, sheep, and goats are invariably corralled at night, often within two concentric, dense thorn-bush walls, with several people nearby. The landscape is unfenced and provides a mosaic of land-use types that allows lions to maintain effective separation from people with access to an adequate prey base. Collectively, these attributes create a system in which lions are stressed by humans and avoid interaction.
